# Supplementary material for: MetSizeR: selecting the optimal sample size for metabolomic studies using an analysis based approach
Source: BMC Bioinformatics. 2013 Nov 21;14:338. doi: 10.1186/1471-2105-14-338 (PMC4222287; doi:10.1186/1471-2105-14-338)
Supplement: Additional file 1 — Simulating from the PPCCA and DPPCA models. [file 1471-2105-14-338-S1.pdf]

Additional file: Simulating from the PPCCA and DPPCA models.

1 The Probabilistic Principal Components and Covariates Analysis (PPCCA) model.

The PPCCA model [1] is an extension of the PPCA model which includes covariates. The PPCCA models the high dimensional spectrum $\underline{x}_i^T = (x_{i1}, \ldots, x_{ip})$ of subject i $(i = 1, \ldots, n)$ as a linear

function of the corresponding low dimensional latent variable $\underline{u}_i^T = (u_{i1}, \ldots, u_{iq})$, where $(q \ll p)$. The PPCCA model can be expressed as follows

$$\underline{x}_i = \mathbf{W}\underline{u}_i + \underline{\mu} + \underline{\epsilon}_i$$

where $\mathbf{W}$ is a $p \times q$ loadings matrix, μ is a mean vector and $\underline{\epsilon}_i$ is multivariate Gaussian noise for observation i, i.e. $p(\underline{\epsilon}_i) = \text{MVN}_p(\underline{0}, \sigma^2\mathbf{I})$ where $\mathbf{I}$ denotes the identity matrix. The PPCCA model differs from the PPCA model in that it allows the covariates to influence the distribution of the latent variables or scores $\mathbf{u}$ i.e.

$$p(\mathbf{u}|\boldsymbol{\beta}) = \prod_{i=1}^{n} \text{MVN}_q(\boldsymbol{\beta}\underline{C}_i, \mathbf{I}).$$

Here $\boldsymbol{\beta}$ is a $q \times (L+1)$ matrix of parameters which capture the relationship between the latent variable and the covariates and $\underline{C}_i$ is a $(L+1)$ vector of an intercept term and the L covariates of observation i.

For a given sample size n, the pilot data can be simulated from the PPCCA model as follows:

1. Generate parameter values from their prior distributions:

$$\begin{aligned} p(\underline{\beta}_k) &= \text{MVN}_{L+1}(\underline{\mu}_\beta, \Sigma_\beta) \text{ for } k = 1, \ldots, q \\ p(\underline{u}_i) &= \text{MVN}_q(\boldsymbol{\beta}\underline{C}_i, \mathbf{I}) \text{ for } i = 1, \ldots, n \\ p(\underline{w}_j) &= \text{MVN}_q(\underline{\mu}_W, \Sigma_W) \text{ for } j = 1, \ldots, p \\ p(\sigma^2) &= \text{IG}[\alpha_1, \alpha_2] \end{aligned}$$

2. Conditional on the generated parameters and latent variables the pilot data $\mathbf{x}$ are then simulated from the PPCCA model:

$$p(\underline{x}_i|\underline{u}_i, \mathbf{W}, \sigma^2) = \text{MVN}_p(\mathbf{W}\underline{u}_i, \sigma^2\mathbf{I})$$

For similar reasons to those discussed in the paper in the case of PPCA, the hyperparameters are specified to be $\underline{\mu}_\beta = \mu_W = 0$, $\Sigma_\beta = \Sigma_W = \mathbf{I}$, $\alpha_1 = 3$ and $\alpha_2 = 4$.

2 The Dynamic Probabilistic Principal Components Analysis (DPPCA) model.

Dynamic PPCA (DPPCA) is another extension of PPCA which allows PPCA to appropriately model longitudinal metabolomic data. The DPPCA model models the correlation due to repeated measurements by assuming a stochastic volatility (SV) model [2] for the errors and for the scores of the PPCA model. As detailed in [3], the error for observation i at time m is assumed distributed as $\underline{\epsilon}_{im} = \text{MVN}_p(\underline{0}, \sigma^2_m \mathbf{I})$ and the associated score is distributed $\underline{u}_{im} = \text{MVN}_q(\underline{0}, \mathbf{H}_m)$, where $\mathbf{H}_m = \text{diag}(h_{1m}, \ldots, h_{qm})$. Under the DPPCA model, at time m, the log volatilities of the errors $\eta_m = \log \sigma^2_m$ and the scores $\lambda_{km} = \log h_{km}$ where $k = 1, \ldots, q$ components, have a stationary autoregressive process AR(1):

$$
\begin{aligned}
\eta_m &= \nu + \phi(\eta_{m-1} - \nu) + r_m \\
\lambda_{km} &= \mu_k + \phi_k(\lambda_{k(m-1)} - \mu) + r_{km}
\end{aligned}
$$

where ν and μ_k are the means and ϕ and ϕ_k are the persistence parameters of the two models respectively. The persistence parameters are constrained between $[-1, 1]$. The innovations of the two models r_m and r_{km} are assumed to be normally distributed, $\text{N}(0, v^2)$ and $\text{N}(0, v^2_k)$ respectively.

Pilot data are simulated from the DPPCA model by focusing on the initial time point of the experiment since it is expected that the same number of subjects are followed over time. The initial state of the DPPCA model, by stationarity, is given as:

$$
p(\mathbf{x}_1 | \mathbf{W}_1, \mathbf{u}_1, \eta_1) = \prod_{i=1}^{n} \text{MVN}_p(\mathbf{W}_1 \underline{u}_{i1}, \exp(\eta_1)\mathbf{I})
$$

where $\mathbf{x}_1, \mathbf{W}_1$ and $\mathbf{u}_1$ are the data, the loadings and the scores at the initial time point. The pilot data for the first time point of a longitudinal metabolomic study are then simulated as follows:

1. Generate the model parameter values and latent variables from their prior distributions:

$$
\begin{aligned}
p(\eta_1 | \nu, \phi, v^2) &= \text{N}\left(\nu, \frac{v^2}{1-\phi^2}\right) \\
p(\lambda_{k1} | \mu_k, \phi_k, v^2_k) &= \text{N}\left(\mu_k, \frac{v^2_k}{1-\phi^2_k}\right) \quad \text{for } k = 1, \ldots, q \\
p(\underline{u}_{i1} | \underline{\lambda}_1) &= \text{MVN}_q(\underline{0}, \mathbf{H}_1) \text{ for } i = 1, \ldots, n \\
p(\underline{w}_{j1}) &= \text{MVN}_q(\underline{\mu}_W, \Sigma_W) \text{ for } j = 1, \ldots, p
\end{aligned}
$$

   where $\mathbf{H}_1 = \text{diag}[\exp(\lambda_{11}), \ldots, \exp(\lambda_{q1})]$.

2. Conditional on the generated parameters and latent variables the pilot data $\mathbf{x}_1$ are then simulated from the DPPCA model:

$$
p(\underline{x}_{i1} | \mathbf{W}_1, \underline{u}_{i1}, \eta_1) = \text{MVN}_p(\mathbf{W}_1 \underline{u}_{i1}, \exp(\eta_1)\mathbf{I}) \text{ for } i = 1, \ldots, n.
$$

   For similar reasons to those discussed in the paper in the case of PPCA, and above in the case of PPCCA, the hyperparameters for the loadings matrix prior distribution are specified to be $\underline{\mu}_W = \underline{0}$ and $\Sigma_W = \mathbf{I}$. For the SV part of the DPPCA model, the hyperparameters are assumed to be: $\nu = \mu_k = 0, \phi = \phi_k = 0.8$, and $v^2 = v^2_k = 0.1$. These values are

based on prior knowledge of longitudinal metabolomic experiments i.e. positive dependence is expected across time and the log volatilities are expected to closely fluctuate around zero.

References

[1] Nyamundanda G, Gormley IC, Brennan L: **Probabilistic principal component analysis for metabolomic data.** *BMC Bioinformatics* 2010, **11**(571).

[2] Kim S, Shephard N, Chibb S: **Stochastic volatility: likelihood inference and comparison with ARCH models**. *Review of economic studies* 1998, **65**:361–393.

[3] Nyamundanda G, Gormley IC, Brennan L: **A dynamic probabilistic principal components model for the analysis of longitudinal metabolomics data.** Tech. rep., University College Dublin 2012.
